# Two Cassava Basic Leucine Zipper (bZIP) Transcription Factors (MebZIP3 and MebZIP5) Confer Disease Resistance against Cassava Bacterial Blight

**DOI:** 10.3389/fpls.2017.02110

**Published:** 2017-12-08

**Authors:** Xiaolin Li, Shuhong Fan, Wei Hu, Guoyin Liu, Yunxie Wei, Chaozu He, Haitao Shi

**Affiliations:** ^1^Hainan Key Laboratory for Sustainable Utilization of Tropical Bioresources and College of Biology, Institute of Tropical Agriculture and Forestry, Hainan University, Haikou, China; ^2^Key Laboratory of Biology and Genetic Resources of Tropical Crops, Institute of Tropical Bioscience and Biotechnology, Chinese Academy of Tropical Agricultural Sciences, Haikou, China

**Keywords:** basic leucine zipper (bZIP) transcription factor, cassava (*Manihot esculenta*), cassava bacterial blight, disease resistance, virus-induced gene silencing (VIGS)

## Abstract

Basic domain-leucine zipper (bZIP) transcription factor, one type of conserved gene family, plays an important role in plant development and stress responses. Although 77 *MebZIPs* have been genome-wide identified in cassava, their *in vivo* roles remain unknown. In this study, we analyzed the expression pattern and the function of two *MebZIPs* (*MebZIP3* and *MebZIP5*) in response to pathogen infection. Gene expression analysis indicated that *MebZIP3* and *MebZIP5* were commonly regulated by flg22, *Xanthomonas axonopodis* pv. *manihotis* (*Xam*), salicylic acid (SA), and hydrogen peroxide (H_2_O_2_). Subcellular localization analysis showed that MebZIP3 and MebZIP5 are specifically located in cell nucleus. Through overexpression in tobacco, we found that *MebZIP3* and *MebZIP5* conferred improved disease resistance against cassava bacterial blight, with more callose depositions. On the contrary, *MebZIP3-* and *MebZIP5*-silenced plants by virus-induced gene silencing (VIGS) showed disease sensitive phenotype, lower transcript levels of defense-related genes and less callose depositions. Taken together, this study highlights the positive role of *MebZIP3* and *MebZIP5* in disease resistance against cassava bacterial blight for further utilization in genetic improvement of cassava disease resistance.

## Introduction

The genus *Xanthomonas* is a kind of plant pathogen that infects a wide range of plant species, including rice, pepper, tomato, citrus, and *Nicotiana benthamiana. Xam* is the causal pathogen of cassava bacterial blight, resulting in leaf wilting, shoot dieback, and stem vascular necrosis (McCallum et al., 2017). Cassava is one major tropical crop; however, its yield is seriously affected by cassava bacterial blight (Pereiral et al., 2003; Camilo et al., 2005; Quintero et al., 2013; Muñoz-Bodnar et al., 2014). To date, the molecular mechanism underlying cassava in response to bacterial blight is largely unknown, and the identification and utilization of disease-related genes are very limited. With the public available cassava genome sequence (Wang et al., 2014), more and more researches start to isolate cassava genes and investigate their role in stress response, starch metabolism, and postharvest physiological deterioration of cassava storage roots (Okogbenin et al., 2013; Xu et al., 2013; Zeng et al., 2014; Zhao et al., 2015; Wei et al., 2016). Although some disease resistant cassava varieties have been identified (Boher and Verdier, 1994; Wydra et al., 2007), functional characterization of disease-related genes remains limited.

The bZIP transcription factor, one type of conserved gene family, plays an important role in plant growth, development, abiotic and biotic stress responses (Alves et al., 2013; E et al., 2014). With the conserved bZIP domain, bZIP family is one of the largest transcription factors in plants. The bZIP domain contains two structural features, a basic region and a leucine zipper (Alagarasan et al., 2017; Zha et al., 2017). The basic region consists of about 16 amino acid residues and an invariant N-x7-R/K motif, which are responsible for nuclear localization and DNA binding, respectively. The leucine zipper includes a heptad repeat of leucines or other bulky hydrophobic amino acids that are positioned exactly nine amino acids toward the C-terminus, forming a superimposing coiled-coil structure (Alves et al., 2013; E et al., 2014). So far, plant bZIP transcription factors preferentially bind to DNA sequences with a core motif of ACGT, such as A-box (TACGTA), C-box (GACGTC), and G-box (CACGTG) (Foster et al., 1994; Sibéril et al., 2001; Jakoby et al., 2002; Schütze et al., 2008).

Through genome-wide analysis, *bZIP* gene family has been identified in numerous plant species, including *Arabidopsis* (Jakoby et al., 2002), pepper (*Capsicum annum*) (Hwang et al., 2005), rice (*Oryza sativa* L.) (Nijhawan et al., 2008), maize (*Zea mays* L.) (Wei et al., 2012), *Populus* (Ji et al., 2013), *Phaseolus vulgaris* (Astudillo et al., 2013), castor bean (*Ricinus communis* L.) (Jin et al., 2014), grapevine (*Vitis vinifera*) (Liu et al., 2014), cucumber (*Cucumis sativus*) (Baloglu et al., 2014), *Brassica rapa* (Hwang et al., 2014), barley (*Hordeum vulgare* L.) (Pourabed et al., 2015), *Brachypodium distachyon* (Liu and Chu, 2015), tomato (*Solanum lycopersicum* L.) (Li et al., 2015), legume (*Lablab purpureus* L.) (Wang et al., 2015), cassava (*Manihot esculenta*) (Hu et al., 2016), apple (*Malus sieversii* L.) (Zhao et al., 2016), and cabbage (*Brassica oleracea*) (Bai et al., 2016). Functional analysis found that plant bZIPs are widely involved in metabolism (Hartmann et al., 2015; Zhang et al., 2015; Sagor et al., 2016), abiotic stress (salt, drought) (Inaba et al., 2015; Moon et al., 2015; Sornaraj et al., 2016; Xu et al., 2016; Zong et al., 2016; Banerjee and Roychoudhury, 2017) and plant–pathogen interaction (Kim and Delaney, 2002; Shearer et al., 2012; Alves et al., 2013, 2015; Lim et al., 2015).

As transcription factors, plant bZIPs regulate down-stream genes through directly binding to their promoter regions (Foster et al., 1994; Sibéril et al., 2001; Jakoby et al., 2002; Schütze et al., 2008). TGA is widely known in plant defense responses. In *Arabidopsis*, TGAs interact with NPR1, and binding to the promoters of SA-responsive genes such as *PR1* (Alves et al., 2013). Moreover, plant bZIPs regulate disease resistance through interacting with other proteins in defense responses, including the interaction of AtbZIP10 and LSD1 (Kaminaka et al., 2006), NtTGAs and NtWRKY12 (van Verk et al., 2011). Although 77 *MebZIPs* have been genome-wide identified in cassava (Hu et al., 2016), their *in vivo* role remains unknown. In this study, the expression pattern and gene function of two *MebZIPs* (*MebZIP3* and *MebZIP5*) in response to pathogen infection were analyzed. We highlight the positive role of *MebZIP3* and *MebZIP5* in disease resistance against cassava bacterial blight for further utilization in genetic improvement of cassava resistance to disease.

## Materials and Methods

### Plant Materials and Growth Conditions

South China 124 variety of *Manihot esculenta* was used. SC124 cassava and tobacco plants were grown in soil with Hoagland’s solution, at 26–28°C, with 12 h light at 120–150 μmol quanta m^-2^ s^-1^ irradiance and 12 h dark cycles.

### RNA Isolation and Quantitative Real-Time PCR

Total RNA extraction and cDNA synthesis were performed from plant leaves using RNAprep Pure Plant Kit (TIANGEN, DP441, Beijing, China) and RevertAid First Strand cDNA Synthesis Kit (Thermo Scientific, K1622, Waltham, MA, United States), according to the manufacturer’s instruction. The quantitative real-time PCR was performed using cDNA and FastStart Essential DNA Green Master (Roche, 06924204001, Basel, Switzerland) and analyzed using the comparative ΔΔ^C_T_^ method as Wei et al. (2016) described. *NtEF1a* and *MeEF1a* were used as internal references for analysis. The primers used for real-time PCR were listed in Supplementary Table S1.

### Vector Construction and Transient Expression in *Nicotiana benthamiana* Leaves

For the vector construction, the coding regions of *MebZIP3* and *MebZIP5* were first amplified by PCR from plant leave samples. Thereafter, the PCR products were cloned into *SpeI* and *NcoI*/*SpeI* digested modified pCAMBIA1302 (Liu et al., 2015) by restriction enzyme digestion and T_4_ ligase ligation, respectively. The primers responsible for vector constructs were listed in Supplementary Table S2, and the restriction enzymes and their cutting sites were marked. The vector cassettes were illustrated in **Supplementary Figure S1**. After DNA sequencing for confirmation, the recombinant plasmids as well as P19 were transformed into *Agrobacterium tumefaciens* strain GV3101. After syringe infiltrating into *Nicotiana benthamiana* leaves as Sparkes et al. (2006) described for 2 dpi, the green fluorescent and DAPI-stained cell nuclei in the infiltrated leaf areas were examined using a confocal laser-scanning microscope (TCS SP8, Leica, Heidelberg, Germany).

### Generation of Transgenic Tobacco Plants

The transgenic *MebZIP3* and *MebZIP5* tobacco plants were generated through *Agrobacterium*-mediated transformation of *MebZIP3*-pCAMBIA1302 and *MebZIP5*-pCAMBIA1302 as Horsch et al. (1985) described. Briefly, 14-day-old sterilized tobacco leaves were incubated in *Agrobacterium* cell suspension for 10 min. Subsequently, the treated leaves were dried with sterilized tissue paper and placed on full MS-Agar medium for co-cultivation. After 2 days, the leaves were transferred to shoot initiation medium with cephalosporin (250 mg L^-1^) and hygromycin (50 mg L^-1^) and the surviving seedlings were grown in a greenhouse to produce seeds for further analysis. The T_2_ transgenic seedlings were selected on MS medium with 50 mg L^-1^ hygromycin, and the green seedlings with long roots were transferred to soil for further semi-quantitative reverse transcriptase-PCR and seed harvest. The transgenic T_3_ seedlings were further selected on MS medium with 50 mg L^-1^ hygromycin to obtain homozygous lines with no segregation on hygromycin resistance, and the independent transgenic T_3_ lines were used for phenotype analysis.

### Virus-Induced Gene Silencing (VIGS) in Cassava

For the vector construction, the partial coding regions of *MebZIP3* and *MebZIP5* were first amplified by PCR from plat leave samples. Thereafter, the PCR products of these genes were cloned into *EcoRI*/*BamHI* digested pTRV2 vector (Liu et al., 2002) by restriction enzyme digestion and T_4_ ligase ligation. The primers that are responsible for vector constructs were listed in Supplementary Table S2, the restriction enzymes and their cutting sites were marked. The vector cassettes were illustrated in **Supplementary Figure S1**. After DNA sequencing for confirmation, the recombinant plasmid (pTRV2-*MebZIP3* and pTRV2-*MebZIP5*) as well as pTRV1 were transformed into *Agrobacterium tumefaciens* strain GV3101. The GV3101 strains were first cultured in 10 ml of LB liquid medium at 28°C for 12 h, and shaken in the new LB liquid culture to reached OD_600_ at about 2. After diluted to OD_600_ of 1 by 10 mM MgCl_2_, 10 mM MES, and 20 mM acetosyringone, the GV3010 strain with pTRV1 and the strain with pTRV2 or *MebZIP3*-pTRV2 or *MebZIP5*-pTRV2 were mixed with ratio of 1:1 and co-infiltrated into cassava leaves as Wei et al. (2017) described. After 14 days, the corresponding gene expression assay and disease resistance assay were performed in plant leaves.

### *Xam* Infection

The bacterial pathogen of *Xam* was first cultured in 10 ml of LB liquid medium at 28°C for 12 h, and shaken in the new LB liquid culture to reach OD_600_ at about 0.6. After diluted to 10^8^ cfu ml^-1^ by 10 mM MgCl_2_ and 0.05% silwet L-77, the *Xam* was syringe infiltrated into abaxial side of plant leaves. Then the plants with pathogen infection were grown in the green house. At indicated time-points, at least 20 leaves were harvested in every biological repeat. Plant leaves were gently washed by sterile distilled water for 1 min, then the bacterial populations in plant leaves were quantified using 10 μl five 10-fold dilutions of homogenate in LB medium.

### Callose Staining

Callose deposition in plant leaves was visualized by callose staining, using alcoholic lactophenol solution, 0.01% (w/v) aniline blue solution, 50% (v/v) glycerol and fluorescence microscope (DM6000B, Leica, Heidelberg, Germany) as Hauck et al. (2003) described.

### Reactive Oxygen Species (ROS) Quantification

The endogenous levels of H_2_O_2_ in plant leaves were extracted and determined using the peroxide-titanium buffer as Wei et al. (2016) previously described.

### Transcriptional Activation Assay in Yeast Cells

For the vector construction, the coding regions of *MebZIP3* and *MebZIP5* were first amplified by PCR from plant leave samples. Thereafter, the PCR products of these were cloned into *NdeI*/*BamHI* and *NcoI*/*BamHI* digested pGBKT7 by restriction enzyme digestion and T_4_ ligase ligation, respectively. The primers that are responsible for vector constructs were listed in Supplementary Table S2, the restriction enzymes and their cutting sites were marked. After DNA sequencing for confirmation, the recombinant plasmids were transformed into yeast strain AH109, according to the manufacturer’s protocol (Clontech, United States). The transformed clones were screened on the SD/-Trp and SD/-His mediums, respectively. The transcriptional activation was evidenced by the growth of yeast cells on SD/-His medium with 5 mM X-α-gal at 30°C for 3 days.

### Accession Numbers

The accession numbers and CDS length of all genes are shown as following: *MebZIP3* (KU160294, 1,788 bp), *MebZIP5* (KU160296, 1,488 bp), *MePR1* (Me07G050300, 492 bp), *MePR2* (Me10G089800, 492 bp), *MePR3* (Me07G050700, 486 bp), *MePR4* (Me07G050400, 492 bp), *MeEF1a* (AF041463, 1,035 bp), *NtEF1a* (AY206004, 661 bp).

### Statistical Analysis

All results in this study were obtained from at least three biological repeats, and the average values and SDs of these biological repeats were shown. In the meanwhile, asterisk symbols (^∗^) indicting the significant differences at *p* < 0.05 were also shown after ANOVA analysis.

## Results

### Expression Profiles of *MebZIP3* and *MebZIP5* in Response to Stress Treatments

In the previous study (Hu et al., 2016), 77 *MebZIPs* have been identified in *Manihot esculenta* Phytozome database v10.3^1^. Herein, a phylogenetic tree between MebIP3/MebZIP5 and their homologs from other plant species were constructed (**Supplementary Figure S2A**), and the results implied the functional similarities among the bZIP proteins in different plants. Moreover, the conserved bZIP domain of MebZIP3 and MebZIP5 was identified (**Supplementary Figure S2B**), further indicating that bZIPs are conserved during evolution.

**FIGURE 1:**
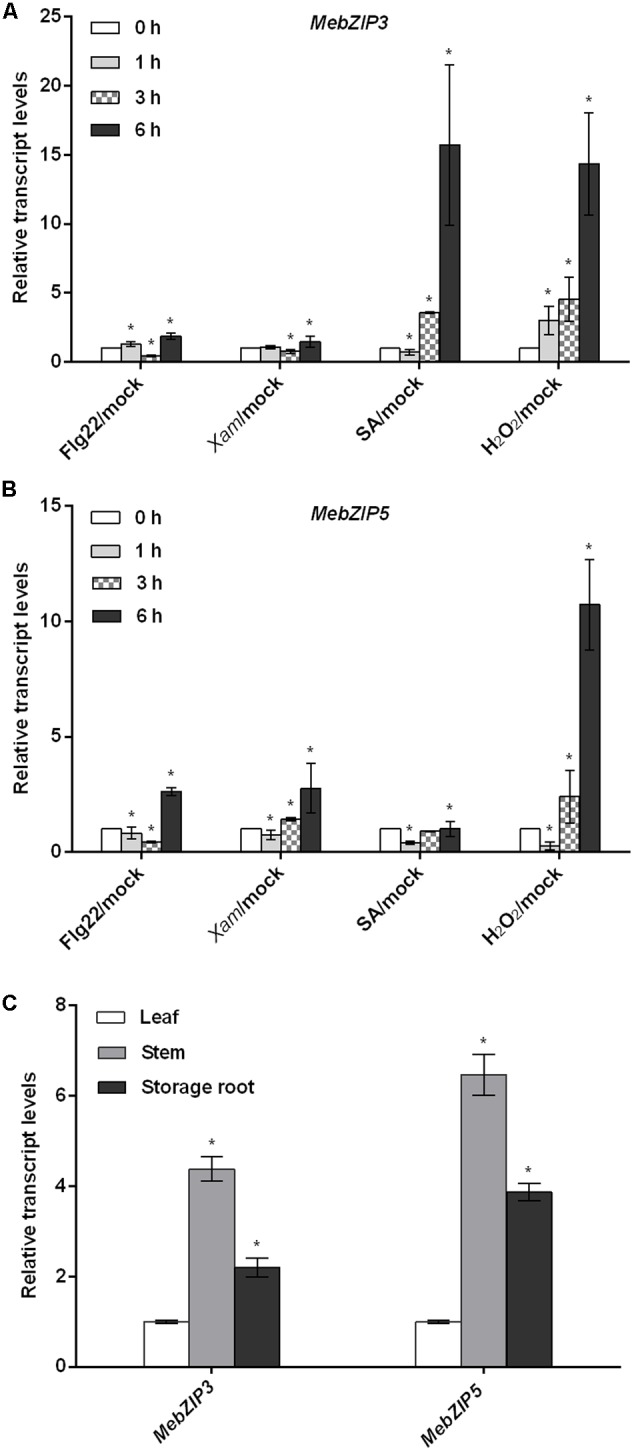
The expression patterns of *MebZIP3* and *MebZIP5*. **(A,B)** The transcript levels of *MebZIP3* **(A)** and *MebZIP5* **(B)** in response to different treatments. For the assay, cassava leaves were sprayed with water (mock), or 10 μM flg22, or 100 μM SA, or 10 mM H_2_O_2_, or syringe infiltrated with 10 mM MgCl_2_ (mock) or 10^8^ cfu ml^-1^ of *Xam* for 0, 1, 3, and 6 h for sample harvest. **(C)** The transcript levels of *MebZIP3* and *MebZIP5* in cassava leaf, stem and storage root. Asterisk symbols (^∗^) indicting the significant differences at *p* < 0.05.

Using quantitative real-time PCR, we found that the transcript levels of *MebZIP3* and *MebZIP5* were significantly regulated after flg22, *Xam*, SA and H_2_O_2_ treatments (**Figures 1A,B**). After flg22 treatment, the transcript levels of *MebZIP3* and *MebZIP5* were down-regulated at 3 h, but largely up-regulated at 6 h. After *Xam* treatment, *MebZIP3* and *MebZIP5* transcripts were significantly induced at 6 h. *MebZIP3* transcript was largely increased after SA treatment for 3 and 6 h, while MebZIP5 expression was decreased after SA treatment for 1 h. Moreover, *MebZIP3* and *MebZIP5* transcripts were largely induced after H_2_O_2_ treatment for 3 and 6 h (**Figures 1A,B**). Generally, the transcripts of *MebZIP3* and *MebZIP5* displayed common expression patterns in response to these treatments, indicating the possible involvement of them in plant disease response. Moreover, we found that *MebZIP3* and *MebZIP5* were expressed in all assayed organs, with higher transcript levels in cassava stem and storage root than in leaf (**Figure 1C**).

### Subcellular Localization of MebZIP3 and MebZIP5

**FIGURE 2:**
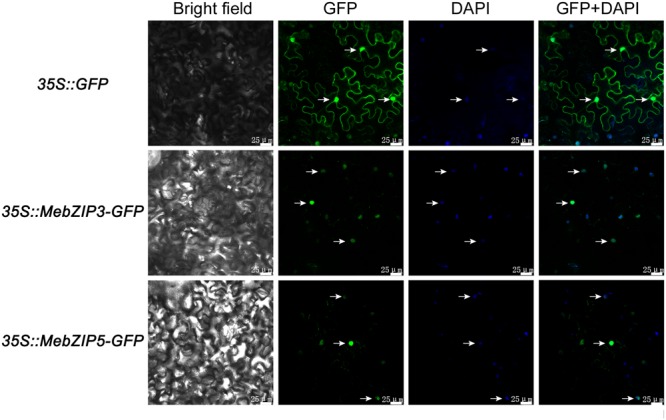
Subcellular localization of MebZIP3 and MebZIP5 in *Nicotiana benthamiana* leaves. After 2 dpi in *Nicotiana benthamiana* leaves, green fluorescent with green color and DAPI-stained cell nuclei with blue color were examined using a confocal laser-scanning microscope. The co-localization of GFP with DAPI was marked by arrow. Bars = 25 μm.

To investigate the subcellular location of MebZIP3 and MebZIP5, the coding regions of these genes were fused with GFP and transiently expressed in *Nicotiana benthamiana* leaves. The control vector (*35S::GFP*)-transformed leaves displayed GFP in both cell nuclei and membrane, consistent with many previous studies (Wei et al., 2017). The GFP signals of MebZIP3-GFP and MebZIP5-GFP were co-localized with DAPI-stained cell nuclei in the infiltrated leaf areas, as marked by the arrow, suggesting that MebZIP3 and MebZIP5 are specifically located in cell nucleus (**Figure 2**).

### Transcriptional Activation Assays of MebZIP3 and MebZIP5

Since bZIPs belongs to transcription factor family, transcription activation assays of MebZIP3 and MebZIP5 were performed in yeast cells. The coding regions of MebZIP3 and MebZIP5 were fused to the GAL4 DNA binding domain in pGBKT7, and the constructs were transformed into yeast strain AH109. As evidenced by the growth of yeast cells and LacZ staining on SD/-His medium with 5 mM X-α-gal, the yeast cells transformed with MebZIP3-pGBKT7 and MebZIP5-pGBKT7 had transcriptional activity (**Supplementary Figure S3**), suggesting the transcriptional activities of MebZIP3 and MebZIP5 in yeast cells.

### Isolation of *MebZIP3* and *MebZIP5* Overexpressing Plants in *Nicotiana benthamiana*

**FIGURE 3:**
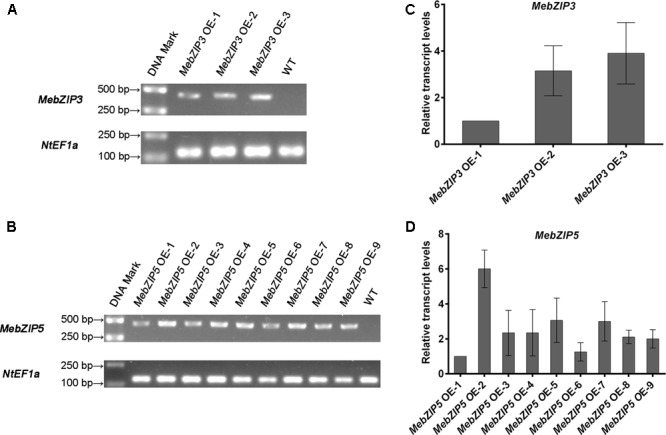
Isolation of *MebZIP3* and *MebZIP5* overexpressing plants in *Nicotiana benthamiana.* **(A,B)** Confirmation of the gene expression in overexpressing lines by semi-quantitative reverse transcriptase-PCR. The expression of *NtEF1a* was used as the internal control. **(C,D)** The relative transcript levels in overexpressing lines by quantitative real-time PCR. Asterisk symbols (^∗^) indicting the significant differences in comparison to mock treatment at *p* < 0.05.

To further reveal the *in vivo* roles of *MebZIP3* and *MebZIP5*, the transgenic plants overexpressing *MebZIP3* or *MebZIP5* were generated in tobacco. After selection on MS medium with hygromycin, the resistant T_1_ transgenic seedlings were transferred to soil, and the gene expressions in the overexpressing lines were confirmed by semi-quantitative reverse transcriptase-PCR (**Figures 3A,B** and **Supplementary Figure S4**). The corresponding *MebZIP3* or *MebZIP5* could be examined in the transgenic *MebZIP3* or *MebZIP5* overexpressing tobacco lines, but could not be amplified in the WT tobacco leaves (**Figures 3A,B** and **Supplementary Figure S4**). The transgenic T_2_ and T_3_ seedlings were further selected on MS medium with 50 mg L^-1^ hygromycin to obtain homozygous lines with no segregation on hygromycin resistance. Because no PCR product was detected in the WT sample by semi-quantitative reverse transcriptase-PCR (**Figures 3A,B**), quantitative real-time PCR was performed to show the relative transcript level in different transgenic T_3_ lines (**Figures 3C,D**). Based on the gene transcript level, three independent transgenic T_3_ lines were used for the phenotype analysis of *MebZIP3* (OE1, OE2, and OE3) and *MebZIP5* (OE2, OE5, and OE7).

### *MebZIP3* and *MebZIP5* Confer Improved Disease Resistance against Cassava Bacterial Blight

**FIGURE 4:**
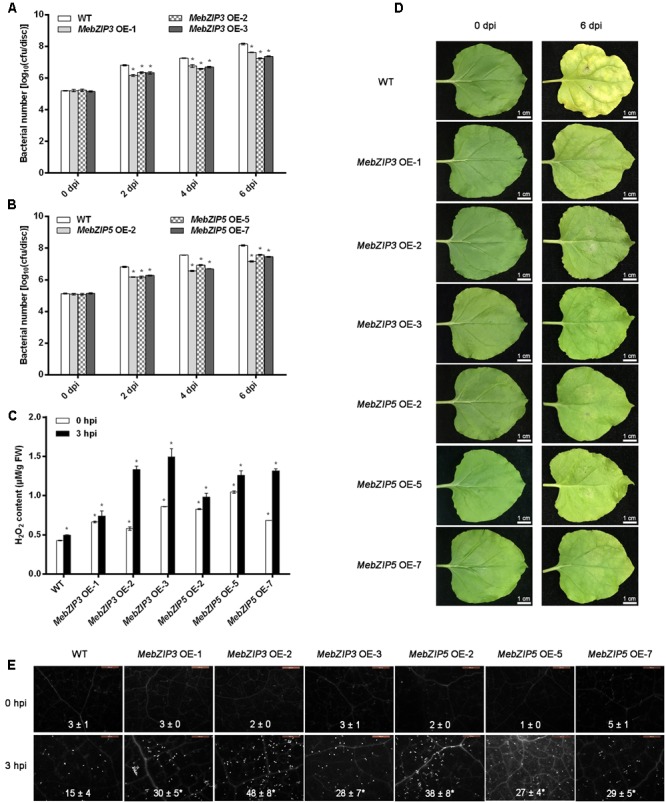
Overexpression of *MebZIP3* and *MebZIP5* confers improved disease resistance against cassava bacterial blight. **(A,B)** The bacterial number of *Xam* in WT, *MebZIP3* **(A)** and *MebZIP5* **(B)** transgenic plant leaves at 0, 2, 4, and 6 dpi of 10^8^ cfu ml^-1^ of *Xam*. **(C)** The pictures showing the plant leaves at 0 and 6 dpi of *Xam*. Bars = 1 cm. **(D)** Quantification of H_2_O_2_ in plant leaves. **(E)** The callose depositions in plant leaves. White dots in the figures indicated callose depositions in plant leaves, and the average data was shown. At least 15 cassava leaves were assayed for every biological repeat, and at least three biological repeats were performed. Asterisk symbols (^∗^) indicting the significant differences in comparison to WT at *p* < 0.05.

Although *Nicotiana benthamiana* is non-host of *Xam*, its leaves can be infected by *Xam* with disease symptom and pathogen growth. To investigate the function of *MebZIP3* and *MebZIP5* in plant disease resistance, the leaf surfaces of WT, *MebZIP3*, and *MebZIP5* transgenic lines were infected with 10^8^ cfu ml^-1^ of *Xam*. At 2, 4, and 6 dpi, three *MebZIP3* (OE1, OE2, and OE3) and three *MebZIP5* (OE2, OE5, and OE7) overexpressing lines exhibited significant less bacterial number in the leaves in comparison to that of WT (**Figures 4A–C**). Moreover, when *Xam* was infected, the H_2_O_2_ and callose depositions were substantially higher in the overexpressing plant leaves than those in WT (**Figures 4D,E**). These results suggested that overexpression of *MebZIP3* and *MebZIP5* conferred improved disease resistance.

**FIGURE 5:**
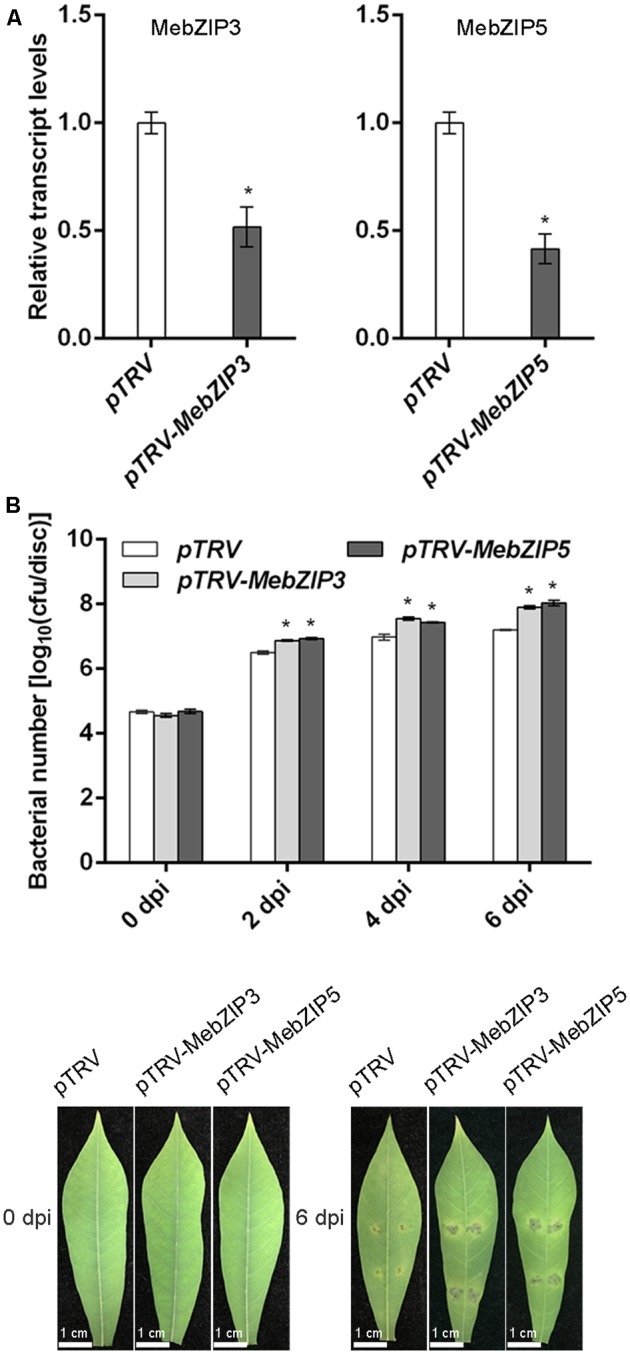
*MebZIP3*- and *MebZIP5*-silenced plants result in disease sensitive. **(A)** The gene transcript levels of *MebZIP3* and *MebZIP5* in the VIGS plants. **(B)** The bacterial number of *Xam* in WT, *MebZIP3-* and *MebZIP5*-silenced plant leaves at 0, 2, 4, and 6 dpi of 10^8^ cfu ml^-1^ of *Xam*. For the assay, *Agrobacterium tumefaciens* strains with the recombinant plasmid (pTRV2-*MebZIP3* and pTRV2-*MebZIP5*) as well as pTRV1 were syringe infiltrated into cassava leaves for 14 days, thereafter the corresponding gene expression assay and disease resistance assay were performed. The cassava leaves were syringe infiltrated by 10^8^ cfu ml^-1^ of *Xam* for another 0, 2, 4, 6 days, and the bacterial number in the cassava leaves were quantified. At least 15 cassava leaves were assayed for every biological repeat, and at least three biological repeats were performed. Asterisk symbols (^∗^) indicting the significant differences in comparison to vector transformation at *p* < 0.05.

**FIGURE 6:**
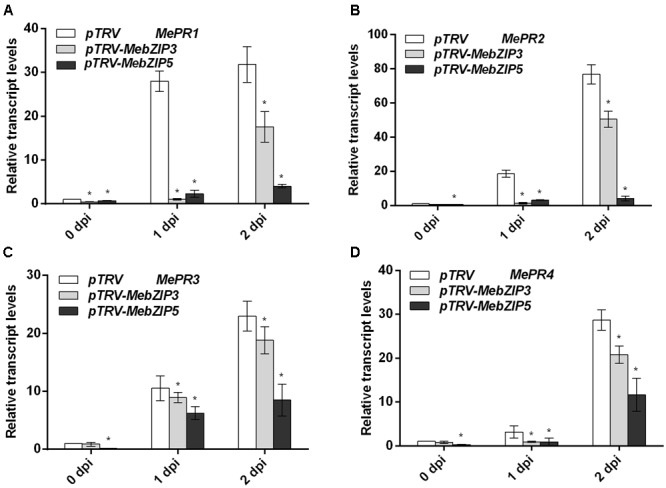
Silencing of *MebZIP3*- and *MebZIP5* regulates the transcripts of defense-related genes. **(A–D)** The relative transcript levels of *MePR1* **(A)**, *MePR2* **(B)**, *MePR3* **(C)**, and *MePR4* **(D)** in the gene silenced plants. At least 15 cassava leaves were assayed for every biological repeat, and at least three biological repeats were performed. Asterisk symbols (^∗^) indicting the significant differences in comparison to vector transformation at *p* < 0.05.

**FIGURE 7:**
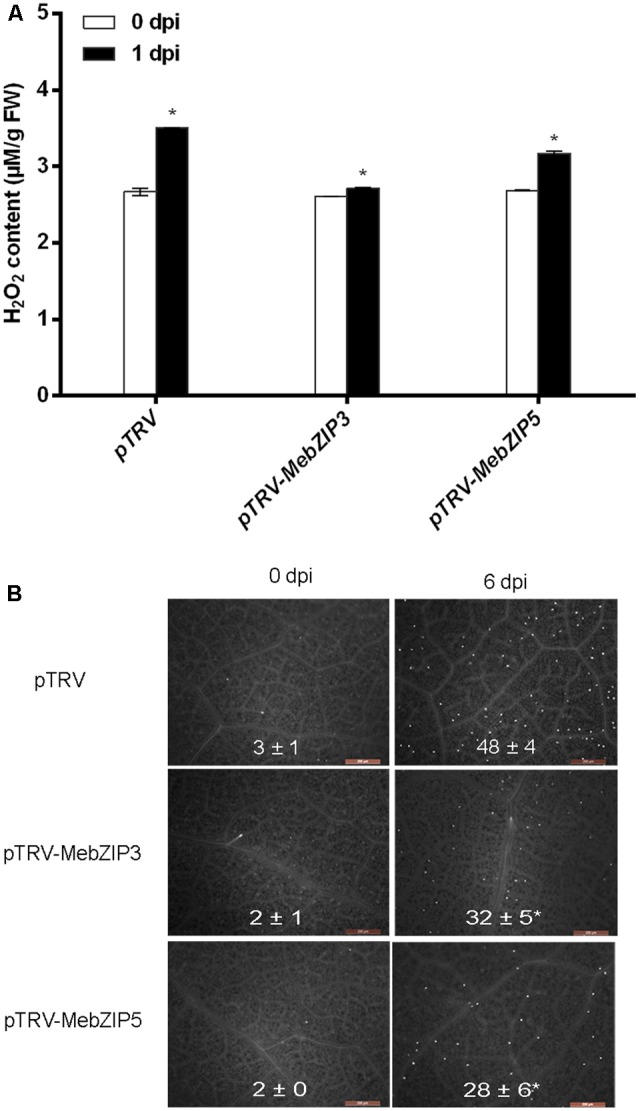
Silencing of *MebZIP3*- and *MebZIP5* regulates callose depositions and ROS level. **(A)** Quantification of H_2_O_2_ in plant leaves. **(B)** The visualization and quantification of calloses in plant leaves. White dots in the figures indicate callose depositions in plant leaves, and the average data was shown. At least 15 cassava leaves were assayed for every biological repeat, and at least three biological repeats were performed. Asterisk symbols (^∗^) indicting the significant differences in comparison to vector transformation at *p* < 0.05.

To further confirm the *in vivo* roles of *MebZIP3* and *MebZIP5* in cassava defense resistance, we construct the *MebZIP3-* and *MebZIP5*-silenced plants through VIGS. As evidenced by the lower transcript of *MebZIP3* or *MebZIP5*, the VIGS plants (*pTRV-MebZIP3* and *pTRV-MebZIP5*) were successfully acquired (**Figure 5A**). In comparison to mock plants, the VIGS plant (*pTRV-MebZIP3* and *pTRV-MebZIP5*) leaves showed more bacterial number (**Figure 5B**), lower transcripts of defense-related genes (*PR1, PR2, PR3*, and *PR4*) (**Figure 6**), less callose depositions and lower levels of H_2_O_2_ in plant leaves upon *Xam* infection (**Figures 7A,B**). Thus, *MebZIP3* and *MebZIP5* are essential for plant disease resistance in cassava.

## Discussion

Although some aquatic plants can move, most plants live as sessile organisms. When subjected to abiotic stress (soil salinity, drought, and extreme temperature) and pathogen infection, plants have to response and cope with these stressors. In the long time of evolution, plants have developed several stress-signaling pathways, including signal receptor, protein kinase, transcription factor, and downstream genes. In the core stress-signaling pathways, transcription factors (including bHLHs, bZIPs, ERFs, ZFPs, WRKYs, MYBs, MYCs) play important roles in linking upstream protein kinase with downstream gene expression (Alves et al., 2013; Okogbenin et al., 2013; E et al., 2014; Hu et al., 2016).

Although 77 *MebZIPs* have been identified in cassava recently (Hu et al., 2016), their *in vivo* roles remain unknown so far. In this study, gene expression analysis showed that *MebZIP3* and *MebZIP5* were commonly regulated by flg22, *Xam*, SA, and H_2_O_2_. With the conserved bZIP domain, transcription activity and specific localization in cell nucleus, MebZIP3 and MebZIP5 are confirmed to be transcription factors. Through overexpression in tobacco, we found that *MebZIP3* and *MebZIP5* conferred improved disease resistance against cassava bacterial blight and more callose depositions. Through VIGS, *MebZIP3-* and *MebZIP5*-silenced plants resulted in disease sensitive, lower transcripts of defense-related genes and less callose depositions. These results are consistent with previous studies that plant bZIP transcription factors are widely involved in plant–pathogen interaction (Kim and Delaney, 2002; Shearer et al., 2012; Alves et al., 2013, 2015; Lim et al., 2015). Thus, we highlight the positive role of *MebZIP3* and *MebZIP5* in disease resistance against cassava bacterial blight for further utilization in genetic improvement of cassava resistance to disease. As reviewed by Alves et al. (2013), TGA is an important bZIP gene in SA signaling. Under control conditions, NPR1 is retained in the cytoplasm as oligomer through *S*-nitrosylation of NPR1 by NO. When the pathogen is infected, SA induces monomeric NPR1 translocates to the nucleus, and NPR1 interacts with TGA family members (bZIPs), and binds to the promoters of SA-responsive genes such as *PR1* (Alves et al., 2013). Although the molecular mechanism of *MebZIP*-mediated defense response remains elusive, the present study provided strong evidence that *MebZIP3* and *MebZIP5* are positive regulators of disease resistance against cassava bacterial blight. Plant bZIPs serve as important regulators of defense resistance through two ways. On one hand, plant bZIPs interact with other proteins in defense responses, including the interaction of AtbZIP10 and AtLSD1 (Kaminaka et al., 2006), AtTGAs and AtNPR1 (Alves et al., 2013), and NtTGAs and NtWRKY12 (van Verk et al., 2011). On the other hand, plant bZIPs preferentially bind to DNA sequences with A-box (TACGTA), C-box (GACGTC), and G-box (CACGTG) (Foster et al., 1994; Sibéril et al., 2001; Jakoby et al., 2002; Schütze et al., 2008). Herein, *MebZIP3-* and *MebZIP5-*silenced plants had significant effects on the transcripts of other *MePRs*, the clone and analysis of *MePRs* promoters will display whether A-box, C-box, and G-box are distributed in these regions. If one of these motifs is distributed in *MePRs* promoters, the underlying *MePRs* may be the direct target of MebZIP3 and MebZIP5. Otherwise, the transcripts of *MePRs* may be affected by MebZIP3 and MebZIP5 indirectly. In further study, the identification of direct targets and interacting proteins of MebZIPs will provide more clues to the underlying mechanism in MebZIPs-mediated defense response in cassava. MebZIP3 and MebZIP5 may interact with other transcription factors to regulate their directly binding to *MePRs*. As a kind of glucan and plant polysaccharide, callose is directly related with callose-associated cell wall and papillae-associated defense (Hauck et al., 2003). Although the underlying mechanism remains unclear, MebZIP3 and MebZIP5-mediated callose accumulation may also contribute to their effects on disease resistance. Taken together, this is the first study showing the positive effects of *MebZIP3* and *MebZIP5* in plant disease resistance against cassava bacterial blight.

## Author Contributions

HS conceived and directed this study, analyzed the data, wrote and revised the manuscript. XL, SF, WH, GL, and YW performed the experiments, analyzed the data, wrote and revised the manuscript. CH provided suggestions and revised the manuscript. All authors approved the manuscript and the version to be published, and agreed to be accountable for all aspects of the work in ensuring that questions related to the accuracy or integrity of any part of the work are appropriately investigated and resolved.

## Conflict of Interest Statement

The authors declare that the research was conducted in the absence of any commercial or financial relationships that could be construed as a potential conflict of interest.

## Footnotes

1 https://phytozome.jgi.doe.gov

## Supplementary Material

FIGURE S1 The vector cassettes in this study.

FIGURE S2 The phylogenetic tree of MebIP3/5 homologs from other plant species. **(B)** The conserved bZIP domain of MebZIP3 and MebZIP5. Multiple sequence alignment and phylogenetic tress were performed by Clustalx 1.83 and MEGA5.05.

FIGURE S3 Transcriptional activation assays of MebZIP3 and MebZIP5 in yeast cells.

FIGURE S4 The original gel images of gene expressions in *MebZIP3* and *MebZIP5* overexpressing lines by semi-quantitative reverse transcriptase-PCR.

## Abbreviations

bHLH: basic helix-loop-helix
bZIP: basic domain-leucine zipper
DAPI: 4′,6-diamidino-2-phenylindole
dpi: days post infiltration
ERFs: ethylene-responsive element-binding factors
GFP: green fluorescent protein
H_2_O_2_: hydrogen peroxide
LSD1: lesions simulating disease resistance 1
NO: nitric oxide
NPR1: non-expresser of PR genes
POX: peroxidase
*PR1*: *pathogenesis-related gene 1*
ROS: reactive oxygen species
SA: salicylic acid
SC124: South China 124
TGA: TGACGTCA *cis*-element-binding protein
VIGS: virus-induced gene silencing
WT: wild type
*Xam*: *Xanthomonas axonopodis* pv. *manihotis*
ZFPs: zinc finger proteins
