# Supplementary figures and images for: Two Cassava Basic Leucine Zipper (bZIP) Transcription Factors (MebZIP3 and MebZIP5) Confer Disease Resistance against Cassava Bacterial Blight
Source: Front Plant Sci. 2017 Dec 8;8:2110. doi: 10.3389/fpls.2017.02110 (PMC5727076; doi:10.3389/fpls.2017.02110)

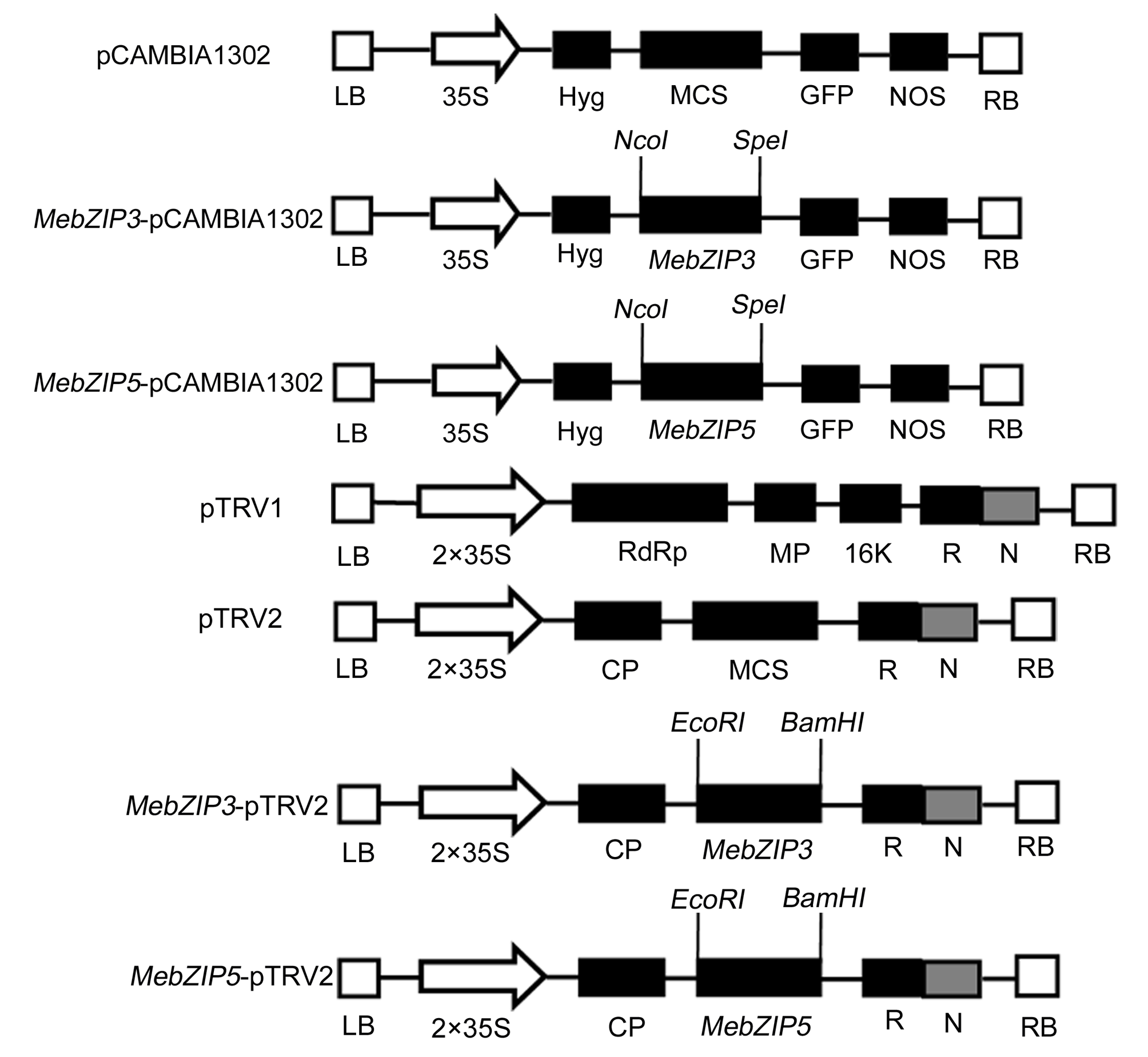

Supplement: FIGURE S1 — The vector cassettes in this study. [file Image_1.TIF]

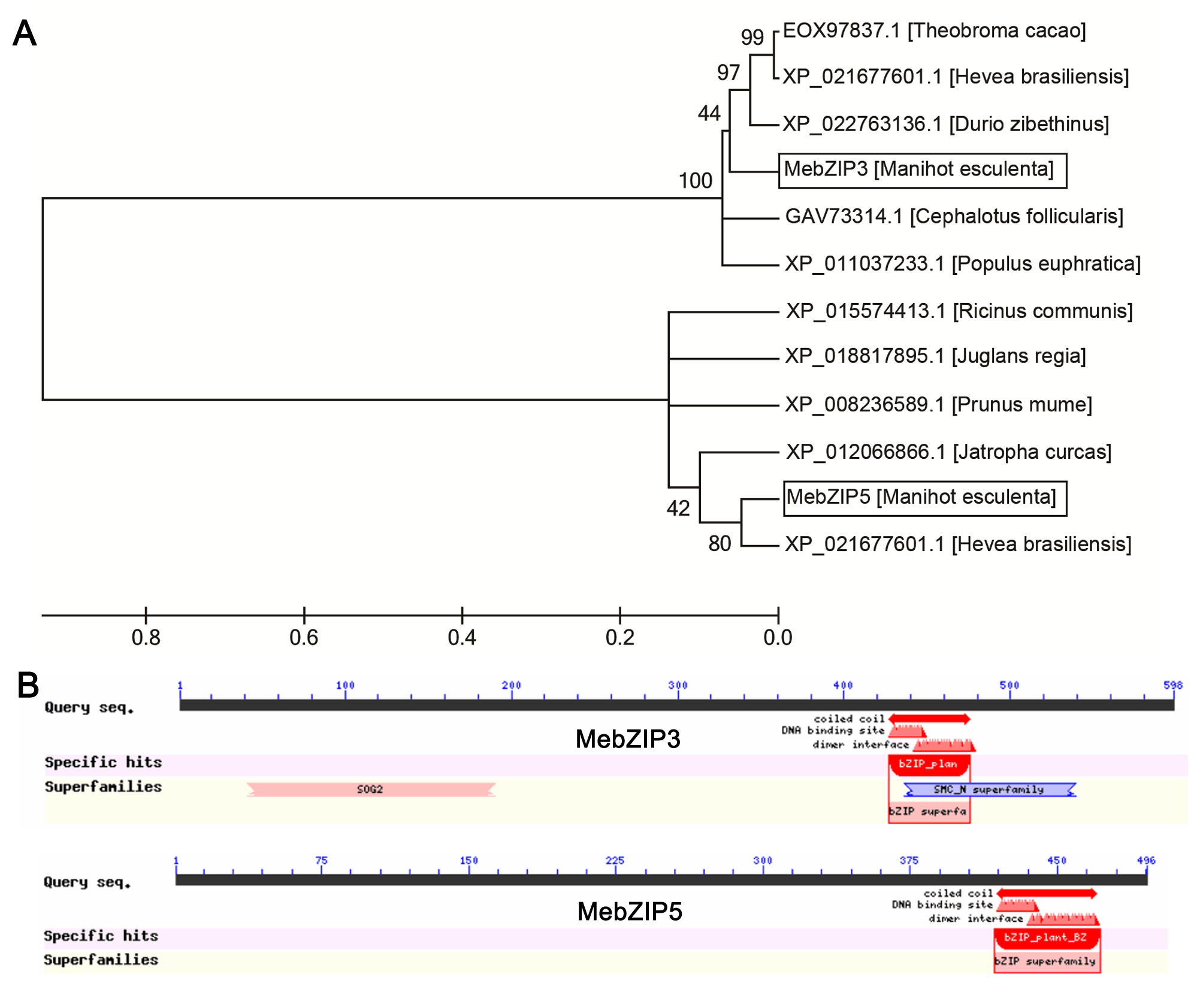

Supplement: FIGURE S2 — The phylogenetic tree of MebIP3/5 homologs from other plant species. (B) The conserved bZIP domain of MebZIP3 and MebZIP5. Multiple sequence alignment and phylogenetic tress were performed by Clustalx 1.83 and MEGA5.05. [file Image_2.TIF]

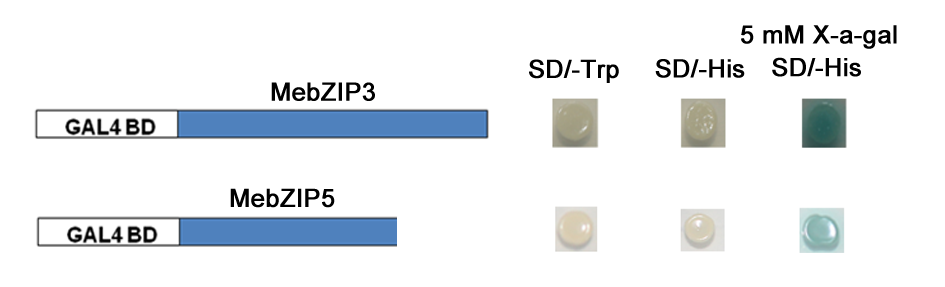

Supplement: FIGURE S3 — Transcriptional activation assays of MebZIP3 and MebZIP5 in yeast cells. [file Image_3.TIF]

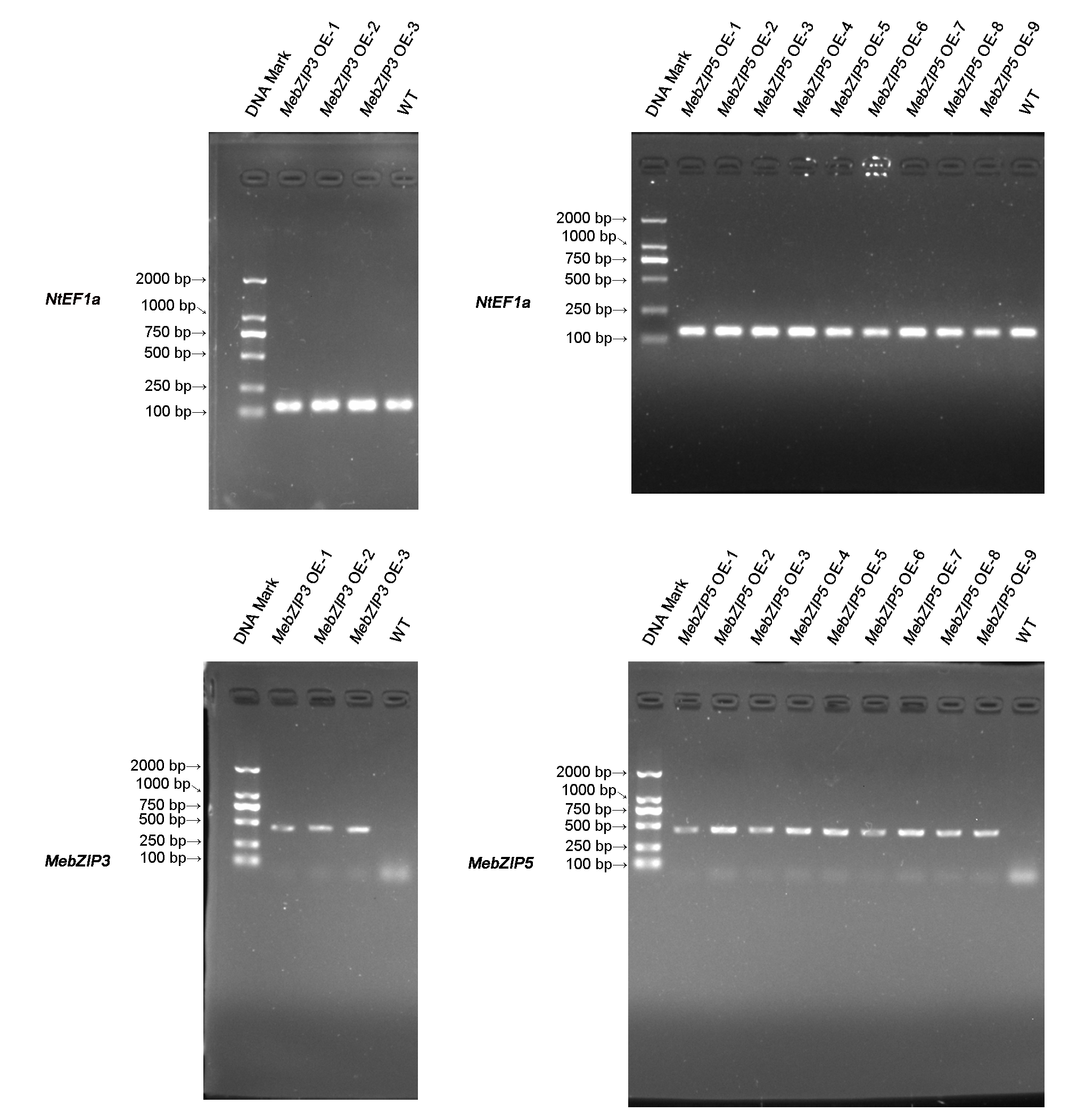

Supplement: FIGURE S4 — The original gel images of gene expressions in MebZIP3 and MebZIP5 overexpressing lines by semi-quantitative reverse transcriptase-PCR. [file Image_4.TIF]
