# Supplementary material for: Two Cassava Basic Leucine Zipper (bZIP) Transcription Factors (MebZIP3 and MebZIP5) Confer Disease Resistance against Cassava Bacterial Blight
Source: Front Plant Sci. 2017 Dec 8;8:2110. doi: 10.3389/fpls.2017.02110 (PMC5727076; doi:10.3389/fpls.2017.02110)
Supplement: Supplementary file 5 [file Table_1.DOC]

**Table S1. The primers used for quantitative real-time PCR.**

| **Gene** | **Primer** | **Sequence** |
| --- | --- | --- |
| *NtEF1a* | QNtEF1aF | AGAGGCCCTCAGACAAAC |
|  | QNtEF1AR | TAGGTCCAAAGGTCACAA |
| *MeEF1a* | QMeEF1aF | TGAACCACCCTGGTCAGATTGGAA |
|  | QMeEF1aR | AACTTGGGCTCCTTCTCAAGCTCT |
| *MebZIP3* | QMebZIP3F | GTGAGTTTAGTGGTGCTG |
|  | QMebZIP3R | AGTAAGCTGGGCAGACAA |
| *MebZIP5* | QMebZIP5F | CAAACAAGCTGGAAGGAG |
|  | QMebZIP5R | ACTGAAGGCTGGCGAATT |
| *MePR1* | QMebPR1F | TCGTCCATTGCCTAAGAT |
|  | QMebPR1R | CGAGTTACGCCAAACCAC |
| *MePR2* | QMebPR2F | AGCATAGCCCTAATAACC |
|  | QMebPR2R | GATAAGTAAATTTCTAAACCCT |
| *MePR3* | QMebPR3F | CCACTATACTCAGGTGGTT |
|  | QMebPR3R | CAAATCTTAGATTGCTTCA |
| *MePR4* | QMebPR4F | ATTATGCCAGTCAACGAG |
|  | QMebPR4R | TTAGTAATGTGAAAGGGAAC |
