# Supplementary material for: Two Cassava Basic Leucine Zipper (bZIP) Transcription Factors (MebZIP3 and MebZIP5) Confer Disease Resistance against Cassava Bacterial Blight
Source: Front Plant Sci. 2017 Dec 8;8:2110. doi: 10.3389/fpls.2017.02110 (PMC5727076; doi:10.3389/fpls.2017.02110)
Supplement: Supplementary file 6 [file Table_2.DOC]

**Table S2. The primers used for vector construction.**

| **Gene** | **Primer** | **Sequence** | **Vector** |
| --- | --- | --- | --- |
| *MebZIP3* | MebZIP3F | GGA’CTAGTATGGGTGATACTGAAGAGGC | pCAMBIA1302 |
|  | MebZIP3R | GGA’CTAGTTTGATTGGACTCTGCATTTG |  |
| *MebZIP5* | MebZIP5F | CATGC’CATGGATGGGATCTCATATGAACTT | pCAMBIA1302 |
|  | MebZIP5R | GGA’CTAGTCCAAGGGCCTGTTAGTGTCC |  |
| *MebZIP3* | MebZIP3F | CCGG’AATTCATGGGTGATACTGAAGAGGC | pTRV2 |
|  | MebZIP3R | CGCG’GATCCACGGAAAGTGGCCGGGCTTA |  |
| *MebZIP5* | MebZIP5F | CCGG’AATTCATGGGATCTCATATGAACTT | pTRV2 |
|  | MebZIP5R | CGCG’GATCCCACATTTGATCCCATGTTGC |  |
| *MebZIP3* | MebZIP3F | GGAATTCCA’TATGATGGGTGATACTGAAGAG | pGBKT7 |
|  | MebZIP3R | CGG’GATCCTCATTGATTGGACTCTGCAT |  |
| *MebZIP5* | MebZIP5F | CATGC’CATGGCGATGGGATCTCATATGAAC | pGBKT7 |
|  | MebZIP5R | CGG’GATCCTCACTTCCCCGGCCAAACATG |  |
